# An Artificial Bee Colony-Based Green Routing Mechanism in WBANs for Sensor-Based E-Healthcare Systems

**DOI:** 10.3390/s18103268

**Published:** 2018-09-28

**Authors:** Jian Yan, Yuhuai Peng, Dawei Shen, Xinxin Yan, Qingxu Deng

**Affiliations:** College of Computer Science and Engineering, Northeastern University, Shenyang 110819, China; jianyan@stumail.neu.edu.cn (J.Y.); shendw@stumail.neu.edu.cn (D.S.); 1310387@stu.neu.edu.cn (X.Y.); dengqx@mail.neu.edu.cn (Q.D.)

**Keywords:** E-Healthcare system, wireless body area networks, energy consumption, Artificial Bee Colony Algorithm

## Abstract

At present, sensor-based E-Healthcare systems are attracting more and more attention from academia and industry. E-Healthcare systems are usually a Wireless Body Area Network (WBANs), which can monitor or diagnose human health by placing miniaturized, low-power sensor nodes in or on patient’s bodies to measure various physiological parameters. However, in this process, WBAN nodes usually use batteries, and especially for implantable flexible nodes, it is difficult to accomplish the battery replacement, so the energy that the node can carry is very limited, making the efficient use of energy the most important problem to consider when designing WBAN routing algorithms. By considering factors such as residual energy of node, the importance level of nodes, path cost and path energy difference ratios, this paper gives a definition of Optimal Path of Energy Consumption (OPEC) in WBANs, and designs the Optimal Energy Consumption routing based on Artificial Bee Colony (ABC) for WBANs (OEABC). A performance simulation is carried out to verify the effectiveness of the OEABC. Simulation results demonstrate that compared with the genetic algorithm and ant colony algorithm, the proposed OEABC has a better energy efficiency and faster convergence rate.

## 1. Introduction

Sensor-based E-Healthcare systems are a new technology developed in the field of health care in recent years. Wireless Body Area Networks (WBANs) [1] are mainly used in emergency rescue, telemedicine, home care and other occasions. Medical sensor nodes are worn on the body surface or implanted in the human body to monitor the patient’s medical data, these physiological data are transferred to a sink through the WBAN, and the sink processes the data or sends the data to a medical monitoring center [2]. The nodes are mostly powered by microbatteries, the energy is very limited, so if some nodes are frequently used for data forwarding, it is likely to lead to the early depletion of these nodes, affecting the network connectivity, and eventually leading to a reduced global network lifetime [3,4,5]. Compared with the traditional data-centric WSN, WBANs’ human-centered features make their energy requirements more demanding [6] is several aspects: (1) Faster signal transmission attenuation: the specificity of the human tissue structure and the shadow effects make the signal in the transmission process have a great path loss [7], resulting in WBANs’ communication energy consumption being much higher than that of other normal networks of the same size. (2) Varying network communication link time: the network topology of WBANs is closely related to the changes in the posture of the human body (especially the activities of the human limbs). The data retransmissions caused by the frequent interruptions of the communication links and the reconstruction of the network topology can cause a waste of energy [8]. (3) Limited node energy: The network lifetime in WBANs is defined as the time interval between when the network starts working to the time when the first node dies, which leads to network partitioning in such a way that the destination cannot be reached. As battery replacement and charging is not feasible in implant medical devices, each node must use limited energy to maximize its own life. Network lifetime is of more importance in WBANs compared to WSNs. Since about 80% of the total energy is consumed only for communication purposes [9], routing algorithms play a vital role to make the communication effective and prolong the lifetime of the WBANs, and how to use the residual energy of nodes effectively to extend the network lifetime becomes the key problem of designing WBAN routing algorithms. In the survey of routing protocols in wireless sensor networks [10,11], the path selection algorithm is based on the principle of minimizing the total energy consumption of the path, the global optimal solution is solved by using the greedy algorithm, which does not consider the protection of individual nodes with small energy, nor the energy consumption ratio of the whole path. It is easy to for some nodes to be utilized with high frequency, and if the important nodes suffer premature death, this often leads to the paralysis of the entire network [12]. Moreover, the time and spatial complexity of these algorithms will usually increase exponentially as the size of the problem grows. In the past decade, the group intelligent optimization methods have attracted more and more attention [13], as they are effective tools to address the problem of combinatorial optimization.

In this paper, we fully consider the energy consumption of the path, the residual energy of the nodes, the importance level of the nodes and the energy ratio of the whole path, and give the definition of Optimal Path of Energy Consumption (OPEC), and by means of the idea of artificial bee colony [14,15,16], design Optimal Energy Consumption Artificial Bee Colony Algorithm-Based WBANs (OEABC). The simulation results show that the OEABC converges faster, and has a better ability to find the optimal solution.

## 2. Related Works

In recent years, several routing protocols have been proposed for WBANs. According to the different routing mechanisms, the energy efficient strategy of WBANs in the network layer is mainly divided into single hop routing, multi-hop routing and cooperative routing. Early research on WBANs mainly focused on the construction of end-to-end network structures, and energy consumption was not a primary concern, so the data was transmitted by using star topologies and single-hop communication modes. The multi-hop routing protocol was proposed and divided into cluster-based routing and cross-layer routing. AnyBody [17], and HIT [18,19] are cluster-based routing protocols that after dividing the nodes into multiple clusters and selecting a cluster head, forward the data to the sink through the cluster head. These protocols aim to minimize the number of direct transmissions from nodes to the sink. However, the large amount of overhead required for cluster selection is the main drawback of these protocols. WASP [20], CICADA [21,22] are cross-layer routing protocols, that by controlling the timing of sending data for every node, lower the energy consumption of WBANs, but require exchanging a lot of control information between nodes. After weighing the advantages and disadvantages of single hop routing and multi-hop routing, collaborative routing [23,24,25] became widely used because it can distribute the transmission load of the whole network. According to the works presented in [23,24], the distance between the source node and sink node can be divided into high and low level. When a source node is located at a high level, multiple hops routing will be used. Otherwise, when it located at a low level, it will choose one hop routing, so the life-cycle of the whole network will be greatly improved. The authors in [25] proposed a decode-and-forward (DF) protocol which uses half duplex mode and builds two communication channels (S-D and S-R-D) between the source node and the sink node. Simulations show this protocol can effectively reduce the energy waste caused by packet retransmission. The pairs of different routing mechanisms are summarized in Table 1.

Certainly, there are many other routing mechanisms. Opportunistic routing [26], PSR [27], PRPL [28], OBSFR [29] are cost-effective routing protocols which select the lowest cost path by monitoring the cost of energy consumption for each path, so the cost-effective relationships between all nodes need to be periodically updated and stored, and a large amount of transmissions and overhead is required to find routes, which also adds complexity to the system. LOCALMOR [30], DMQOS [31] are based on QoS [32] routing protocols, according to the data type collected by the node to select different path metrics to meet the node’s demand for data transmission reliability, delay and energy consumption, but QoS routing protocols require too much information that leads to high energy consumption and huge overhead. TARA [33], LTR [34], LTRT [35] are temperature-based protocols, which are mainly designed to minimize the local or overall system temperature rise. The idea behind these protocols is to route data along different paths to avoid a dramatic temperature rise in some nodes leading to human tissue damage and depletion of the node. In fact, each classification of routing protocols only tries to satisfy a specific requirement in WBANs. Moreover, when choosing a path, the global network lifetime [36] is considered to be insufficient, and the greedy algorithm is used when selecting the path, which easily falls into a local optimum.

## 3. Network Model

### 3.1. Mathematical Description of WBANs

**Definition** **1.** *The set*W={V,C,α,E,A}*represents WBANs, where*$V=\{v_{0},v_{1},v_{2},\ldots ,v_{n}\}$*is the set of nodes;*$C=\{c_{0},c_{1},c_{2},\ldots ,c_{n}\}$*is the set of residual energy of nodes,*$c_{i}\geq 1$*;*$\alpha =\{\alpha_{0},\alpha_{1},\alpha_{2},\ldots ,\alpha_{n}\}$*is the set of importance level of nodes,*α∈{1,2,3,4,5,6,7,8,9}*;*$E=\{e_{0},e_{1},e_{2},\ldots ,e_{m}\}$*is the edge sets,*E⊆{<i,j>|i,j=0,1,2,…,n}*;*A*is the adjacency matrix of nodes, when*<i,j>∈E*,*$a_{ij}$*represents the energy required to transmit a packet from node i to node j, when*<i,j>∉E*,*$a_{ij}=0$.

### 3.2. The Important Level Factor α

There are many types of medical sensor collection activities. Some are essential, and must always be guaranteed, such as ECGs or blood pressure; some can be experienced interruption or delay, such as body temperature; and some nodes even only have forwarding functions, without the acquisition function. According to these characteristics, the importance level factor *α* of the node is defined, so the higher the importance level of the node, the greater the factor *α*. The maximum *α* is 9, and the lowest is 1.

**Figure 1:**
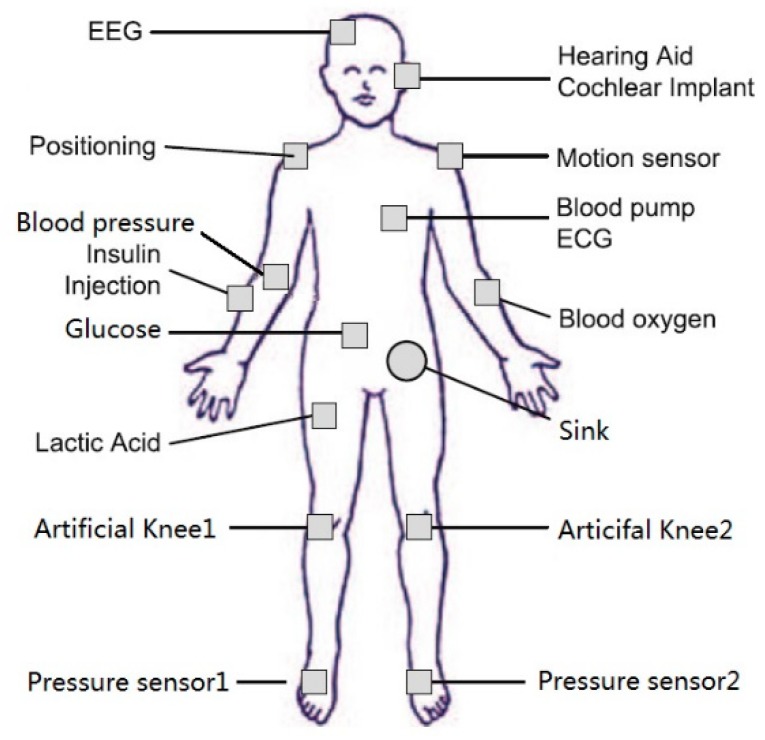
Distributed sample of wireless body area network sensors.

**Figure 2:**
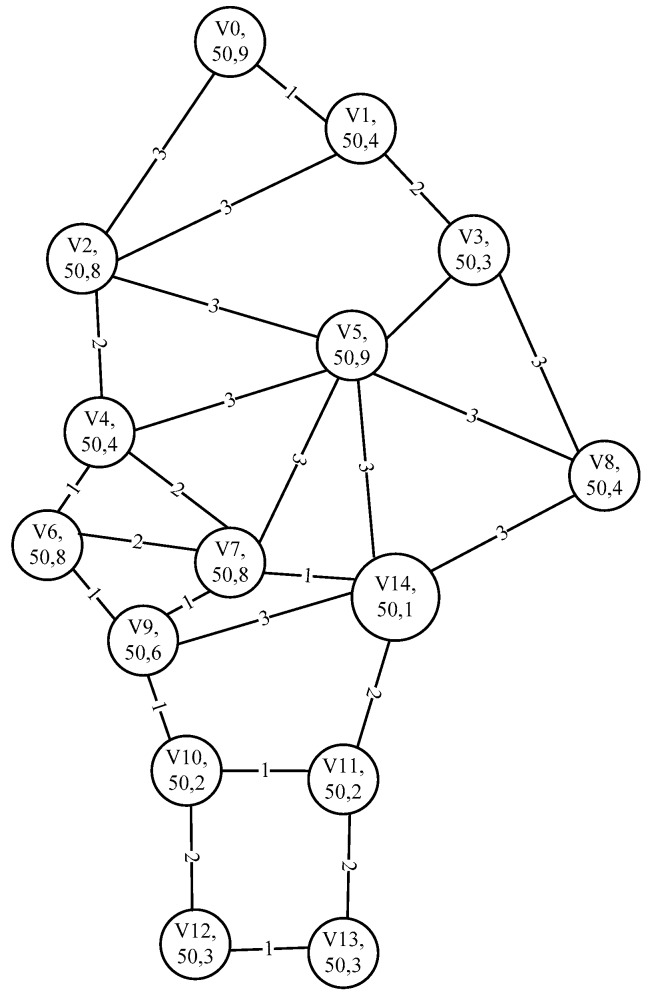
The network topology of Figure 1.

In Figure 1 [29], the WBANs has 15 various types of nodes, the node set *V* is {*v*_0_, *v*_1_, *v*_2_, *v*_3_, *v*_4_, *v*_5_, *v*_6_, *v*_7_, *v*_8_, *v*_9_, *v*_10_, *v*_11_, *v*_12_, *v*_13_, *v*_14_}, where *v*_0_ is EEG, *v*_1_ is hearing aid cochlear implant, *v*_2_ is positioning, *v*_3_ is motion sensor, *v*_4_ is blood pressure, *v*_5_ is Blood pump ECG, *v*_6_ is insulin injection, *v*_7_ is glucose, *v*_8_ is blood oxygen, *v*_9_ is lactic acid, *v*_10_ is artificial knee1, *v*_11_ is artificial knee2, *v*_12_ is pressure sensor1, *v*_13_ is pressure sensor2, *v*_14_ is sink [30]. The initial value of the residual energy of all nodes is 50, the node’s important level factor *α* varies with the sensor node, and the corresponding network topology as shown in Figure 2, where each node includes the node name, the residual energy and the importance level factor *α*. The number on the link between nodes is the transmission energy consumption.

## 4. The Optimal Path of Energy Consumption (OPEC)

### 4.1. Mathematical Description of Path Energy Consumption

**Definition** **2.** *Path Set, denoted as P. The source node is v_0_, the destination node is v_n_, the alternate sequence v_0_…v_i_v_j_…v_n_ is a path from v_0_ to v_n_, let P= {r_1_, r_2_, r_3_,…, r_k_}, so P is the collection of all paths from v_0_ to v_n_.*

**Definition** **3.** *Path Transmission Energy Consumption, denoted as E. The power consumption required to transfer a packet through r_i_ is formulated as:*

(1) $$E(r_{i})=\sum_{0}^{n}a_{ij}$$

The larger the *E*(*r_i_*), the greater the energy required to transfer the data using the path, and vice versa.

**Definition** **4.** *Path Cost, denoted as C. The cost of the node v_i_ is formulated as:*

(2) $$f(v_{i})=\frac{1}{c_{i}}$$

The cost of the path *r_i_* is the sum of the cost of all nodes in the path and is formulated as:

(3) $$C(r_{i})=\sum_{i=0}^{n}f(v_{i})$$

With the decrease of the residual energy of the node, the cost of transmitting the data of the node will be increased, the *C*(*r_i_*) when the path *r_i_* containing the node *v_i_* also increases, and vice versa.

**Definition** **5.** *Path Energy Difference Ratio, denoted as η. In the nodes of path r_i_, the node with the largest remaining energy is max and the node with the smallest energy is min, the path r_i_ energy difference ratio η is formulated as:*

(4) $$\eta (r_{i})=\frac{c_{\min}}{\alpha_{\min}\times c_{\max}}$$

*η*(*r_i_*) indicates the degree of energy difference between the maximum energy node *v_max_* and the minimum node *v_min_* in the path *r_i_*, and the larger the *η*, the smaller the difference is, the better the energy consumption of the path *r_i_*, otherwise the worse. *α*_min_ is the importance level factor of *v_min_*, used to adjust *η*, when *v_min_* is very important or the energy will be exhausted, you can increase *α*_min_, making *η* smaller. At the same time, in order to protect the node which residual energy has reached its may easily cause some nodes to run out of energy, the path will not be utilized.

### 4.2. Definition of the OPEC

The OPEC is denoted by *r_opt_*, choosing *r_opt_* can use the energy of nodes reasonably and efficiently, so that the energy of the whole network can be steadily reduced and the survival time of the whole network can be prolonged. Therefore, when calculating *r_opt_* we need to consider the energy difference ratio *η*, path cost *C* and transmission energy *E*, but the weight of each parameter is different. First we consider *η*, select the maximum path of *η* from all paths that conform to *η_r_* > *η*_Th_, and try not to use less energy or very important nodes to transmit data. If there are multiple paths with the same *η*, the path with the lowest *C* is selected, to reduce the usage of nodes with less residual energy. If there are still multiple paths with the same *η* and *C*, select the path with the lowest *E*.

**Definition** **6.** *Calculation equation of the OPEC:*

(5) $$r_{opt}=\left\{ \begin{array}{l} \max(\eta (r_{i})|\eta_{r_{1}}\neq \eta_{r_{2}}\neq \ldots \neq \eta_{r_{k}})\\ \min(C(r_{i})|\eta_{r_{1}}=\eta_{r_{2}}=\ldots =\eta_{r_{k}})\\ \min(E(r_{i})|(\eta_{r_{1}}=\eta_{r_{2}}=\ldots =\eta_{r_{k}})||(C_{r_{1}}=C_{r_{2}}=\ldots =C_{r_{k}})) \end{array}\right.$$

## 5. Introduction to the Artificial Bee Colony (ABC) Algorithm

The ABC algorithm is inspired by the behavior of bee colonies, which is an optimization method mimicking the behavior of bees, proposed by the Karaboga team in 2005 to optimize the algebra problem [14]. It is an application of cluster intelligence concepts. Through the local optimization of artificial bees, the global optimal value emerges. In the ABC algorithm, the colony of artificial bees contains three groups of bees: worker bees, onlookers and scouts. A bee waiting on the dance area for making decision to choose a food source, is called an onlooker and a bee going to the food source visited by itself previously is named a worker bee, a bee carrying out random search is called a scout. The position of a food source represents a possible solution of the optimization problem and the amount of nectar of a food source corresponds to the quality (fitness) of the associated solution. In the first step, the ABC generates a randomly distributed initial population of *SN* solutions, where *SN* denotes the size of the population. Each solution *X_i_* (*i* = 1, 2, …, *SN*) is a D-dimensional vector. Here, D is the number of optimization parameters. In order to produce an initial food position, the ABC uses the following expression:

(6) $$x_{i,j}=x_{\min,j}+rand(0,1)(x_{\max,j}-x_{\min,j})$$

where *x*_max_ and *x*_min_ are the upper and lower bounds of search space.

After initialization, the worker bee will start the neighborhood search. It generates a new candidate position based on the local information in its memory and checks the amount of nectar in the new location. If the new location is better than the original position, the bee remembers the new location and forgets the original location. The new candidate position equation is:

(7) $$v_{i,j}=x_{i,j}+\phi_{i,j}(x_{i,j}-x_{k,j})$$

The above is called the ABC algorithm search equation, where k∈{1,2,…,BN}, j∈{1,2,…,D} are randomly chosen indexes. Although *k* is determined randomly, it has to be different from *i*. $\phi_{i,j}$ is a random number between [−1, 1].

After the worker bees complete the search process, they will share the memory of the nectar with onlookers through the dance. An onlooker bee chooses a food source depending on the probability *p_i_* associated with that food source, *p_i_* is calculated by the following expression:

(8) $$p_{i}=\frac{fit_{i}}{\sum_{j=1}^{SN}fit_{j}}$$

where $fit_{i}$ is the fitness value of the solution *i* evaluated by its employed bee. When a nectar source is selected, the onlooker bee will use Equation (8) to produce a new position. By checking the amount of nectar in the new position, if the position is better than the one in memory, it will be replaced, otherwise the original position is left. In order to prevent the algorithm from falling into a local optimum, if the amount of nectar does not improve after the limit cycle, the colony will abandon the position, whereby the worker bee becomes a scout and randomly generates a new nectar according to Equation (7).

## 6. The Optimal Energy Consumption Artificial Bee Colony-Based WBAN (OEABC)

### 6.1. The Fitness Function of the Path

The general algebra problem can construct the fitness function directly according to the requirement of the problem. It can be found that the optimal path of energy needs to consider the Path Energy Difference Ratio *η*, Path Cost *C*, Path Transmission Energy Consumption *E*, so the higher the fitness of the path, the better the energy consumption. We define the fitness of the path *r_k_* according to Equation (9):

(9) $$f(r_{k})=\alpha \times \eta (r_{k})+\frac{\beta }{C(r_{k})}+\gamma \times E(r_{k})$$

where α,β,γ are the adjustment parameters to ensure that the *η* is the maximum weight, followed by *C*. *E* is minimum, and its value depends on the specific network environment.

### 6.2. Initial Path Generation Formula

In the initial stage, the ABC algorithm generates *SN* initial solutions for the worker bees randomly by using Equation (6). In the algebraic optimization problem, the solution space is continuous, so the randomly generated solutions are feasible solutions, but each solution of the path planning problem is a path in a particular network topology, its path is discrete, so not any combination of nodes is a path, therefore, we cannot directly apply Equation (6) to generate the initial path.

In order to find a random path, our method is to randomly select a node from neighbors of node *v*_0_ as the next node of the path, and then continue to generate the next node of the path in the same way. If one cannot find the neighbor node, then one goes back to the previous step to select another neighbor node as the next node until the next node is the path end point *v_n_*, to obtain a random path.

Let the function *Adj*(*i*) return the set of all adjacencies of node *i*. Then Equation (10) for generating the node *t* in path *r_i_* is:

(10) $$r_{i,t}=Rand(Adj(t-1))$$

where *t*∈{0, 1, …, *m* − 1}, *m* is the number of nodes of path *i*, *t* is a randomly selected node in path *i*, and *t* − 1 is the predecessor node of node t.

### 6.3. The Generation Formula of the New Path

**Definition** **7.** *In two paths, if there are sections which have the same starting and ending points, it is said that same segments are Same Area (SA) for the two paths.*

**Conclusion.**  $r_{i}=<v_{0}\ldots v_{r}\ldots v_{s}\ldots v_{n}>$$r_{j}=<v_{0}\ldots v_{p}\ldots v_{q}\ldots v_{n}>$, *r_i_ and r_j_ have multiple SA, randomly select an SA, denoted as SA_α_. SA_α_ in r_i_ is <r … s>, denoted as SA_αi_; in r_j_ is <p … q>, denoted as SA_αj_. If SA_αi_ in path r_i_ is replaced by SA_αj_, so the new path is*$<v_{0}\ldots v_{r-1},v_{p}\ldots v_{q},v_{s+1}\ldots v_{n}>$*and is still connected*.

**Proof.**  *r_i_* is a connected path, and its sub-paths $<v_{0}\ldots v_{r-1}>$ and $<v_{s+1}\ldots v_{n}>$ are also connected in accordance with the characteristics of the connected path.

Using the *SA_αj_* of the *r_j_* path to replace the *SA_αi_* of the *r_i_* path, the *r_i_* becomes $<v_{0}\ldots v_{r-1},v_{p}\ldots v_{q},v_{s+1}\ldots v_{n}>$, denoted as ${r_{i}}^{*}$.

p=r, then $<v_{0}\ldots v_{r-1},v_{p}>$ is connected.

q=s, then $<v_{q},v_{s+1}\ldots v_{n}>$ is connected.

Since *r_j_* is connected, so the $SA<v_{p}\ldots v_{q}>$ of its must be connected.

In summary, the sub-paths $<v_{0}\ldots v_{r-1},v_{p}>$, $<v_{p}\ldots v_{q}>$, $<v_{q},v_{s+1}\ldots v_{n}>$ are connected, so their order combination ${r_{i}}^{*}=<v_{0}\ldots v_{r-1},v_{p}\ldots v_{q},v_{s+1}\ldots v_{n}>$ must be connected.     □

When path *i* and *j* exist *k SA*, then $SA_{t}$ in the path *i* and *j* are denoted as $SA_{ti}$ and $SA_{tj}$ respectively, so path $i=<v_{0}\ldots v_{r-1}>SA_{ti}<v_{s+1}\ldots v_{n}>$. The function GetSA(i,j,t) returns $SA_{tj}$, then the equation for generating the new path ${r_{i}}^{*}$ is:

(11) $${r_{i}}^{*}=\left\{ \begin{array}{l} <v_{0}\ldots v_{r-1}>GetSA(i,j,t)<v_{s+1}\ldots v_{n}>\\ r_{i}(when\;r_{i}\;and\;r_{j}\;have\;no\;SA) \end{array}\right.$$

Equation (11) is the search equation for the OEABC algorithm, i,j∈{1,2,…,SN}, t∈{0,1,…,m−1}, *m* is the number of *SA* of path *i* and path *j*.

**Figure 3:**
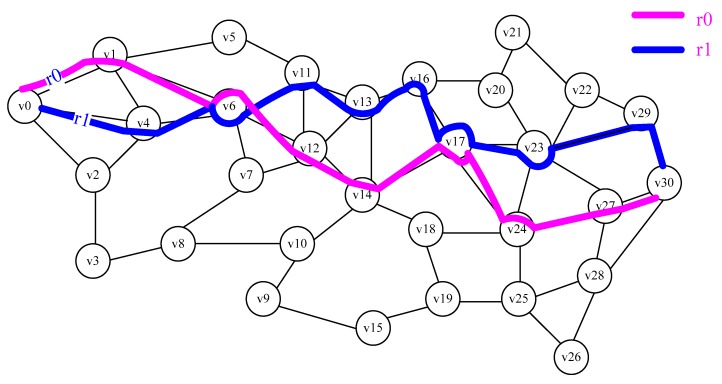
Two paths of r_0_ and r_1_.

**Figure 4:**
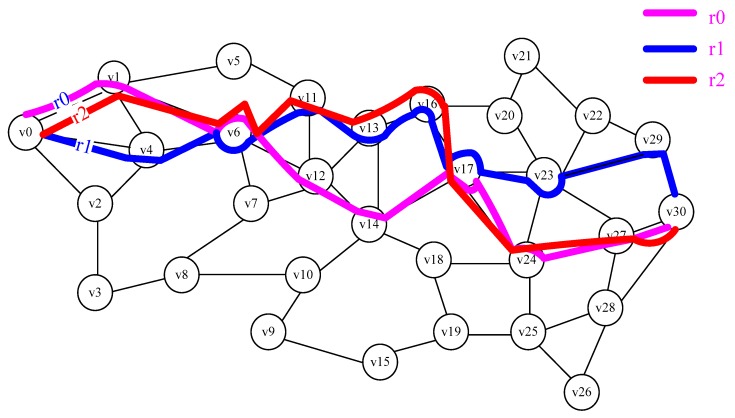
The new paths r_2_ generated by r_0_.

For example, the WBANs with 31 nodes, v_0_ is the starting node, *v*_30_ is the sink, there are already two paths *r*_0_ and *r*_1_, *r*_0_ = <*v*_0_, *v*_1_, *v*_6_, *v*_12_, *v*_14_, *v*_17_, *v*_24_, *v*_27_, *v*_30_>, *r*_1_ = <*v*_0_, *v*_4_, *v*_6_, *v*_11_, *v*_13_, *v*_16_, *v*_17_, *v*_23_, *v*_29_, *v*_30_>, as shown in Figure 3. According to Definition 7, there are 5 *SAs* for *r*_0_ and *r*_1_, in *r*_0_ is <*v*_0_, *v*_1_, *v*_6_>, <*v*_0_, *v*_1_, *v*_6_, *v*_12_, *v*_14_, *v*_17_>, <*v*_6_, *v*_12_, *v*_14_, *v*_17_>, <*v*_6_, *v*_12_, *v*_14_, *v*_17_, *v*_24_, *v*_27_, *v*_30_>, <*v*_17_, *v*_24_, *v*_27_, *v*_30_>, in *r*_1_ is <*v*_0_, *v*_4_, *v*_6_>, <*v*_0_, *v*_4_, *v*_6_, *v*_11_, *v*_13_, *v*_16_, *v*_17_>, <*v*_6_, *v*_11_, *v*_13_, *v*_16_, *v*_17_>, <*v*_6_, *v*_11_, *v*_13_, *v*_16_, *v*_17_, *v*_23_, *v*_29_, *v*_30_>, <*v*_17_, *v*_23_, *v*_29_, *v*_30_>. If *t* = 2, then *GetSA*(*i*, *j*, *t*) = <*v*_6_, *v*_11_, *v*_13_, *v*_16_, *v*_17_>, according to Equation (11), the new paths *r*_2_ = <*v*_0_, *v*_1_, *v*_6_, *v*_11_, *v*_13_, *v*_16_, *v*_17_, *v*_24_, *v*_27_, *v*_30_> generated by *r*_0_, as shown in Figure 4.

OEABC is implemented using the following algorithm (Algorithm 1):


**Algorithm 1. OEABC Algorithm**
**Input:** Network topology, starting point, end point **Output:** Optimal energy consumption pathGenerate the data structure of the WBANs’ topology;Initialize the population of bees according to Equation (10), generate the initial path of *SN*;**For** (num = 0→maxCycle){ The employed bee search better path[i] in the neighborhood according to Equation (11); Calculate the fitness[i] of path[i] according to Equation (9); Using prob[i] = (0.9*(fitness[i]/maxfit)) + 0.1, calculate the selection probability of path[i]; **For** (*t* = 0→SN){  randvalue = ((double)rand()/((double)(RAND_MAX) + (double)(1)));  **If** randvalue < prob[i] **Then**   t++;   r = ((double)rand()/((double)(RAND_MAX) + (double)(1)));   neighbor = (int)(r*FoodNumber);   **While** (neighbour==i){    r = ((double)rand()/((double)(RAND_MAX)+(double)(1)));    neighbor = (int)(r*FoodNumber);}   The onlooker produce new path between path[i] and path[neighbour] according to Equation (11);   Calculate the fitness of the path[new] according to Equation (9);   **If** fitness[new] > fitness[i] **Then**    path[i] = path[new];}   **End If**  **End If** } **If** path[i] is still not optimized by the maxtrial round search **Then**
  send ScoutBees, using Equation (10) to reinitialize path[i]; **End If**}

## 7. Experiments and Results Analysis

We set the body area network node set *V* = {*v*_0_, *v*_2_, *v*_3_, …, *v*_150_} for the simulation environment, the data sending node is *v*_0_, and the sink node is *v*_150_; the node remaining energy *c_i_* ∊ {1, 2, …, 50}; the node service factor *α_i_* ∊ {1, 2, 3}; *E* = {*e*_1_, *e*_2_, *e*_3_, …, *e*_1525_} is an edge set. When <*i*, *j*> ∊ *E*, node *i* transmits a packet to node *j*, energy consumption *a_ij_* ∊ {1, 2, 3}. In order to facilitate the verification result, it is assumed that the node topology is a hierarchical network, and each layer node is only connected to the upper layer and the next layer. The adjustment parameters for setting the fitness function are: *α* = 50, *β* = 5, *γ* = 0.1.

We implemented the OEABC algorithm in C++ programming and tested it in the above simulation environment. In our simulation configuration, we adopt the following simulation settings: the data collection node is *v*_0_ and the sink node is *v*_150_. The communication link is established between neighbor nodes, and each node has 10–15 predecessors and successors. The data packet is sent out from *v*_0_, the data packet is exchanged by the successor node, the transmission path from the data collection node to the network sink node is established, and the data packet is sent to the sink node *v*_150_. The main simulation parameter values are listed in Table 2 below.

### 7.1. Fitness Analysis of the Path

The OEABC parameters are set as: *SN* = 5, *Limit* = 10, *Cycle* = 50, and the running process of the algorithm is as follows:

The first step is to initialize the population. Randomly generate five paths and calculate the fitness of each path. The results are shown in Table 3. Path 4 has the best fitness and is the optimal path.

The second stage is the worker bee stage. The results are shown in Table 4. It can be seen that paths 1 and 4 are optimized, the path fitness is improved, and the optimal path is path 4.

The third stage is the onlooker stage. As shown in Table 5, path 3 is optimized and the optimal path is still path 4. Since it is the first operation of the algorithm, it does not meet the requirements of Limit, so the scout phase is not performed.

The results of the operations after 50 iterations, are shown in Table 6. It can be seen that the five paths have been optimized, the optimal path has changed from path 4 to path 3, and the fitness has also increased from the initial 58.42 to 71.46.

### 7.2. Parameter Analysis

Before running the OEABC, one needs to set the *SN*, *Limit*, and *Cycle* parameters. *SN* is the population size. *Limit* is when to give up with the current solution required for the maximum number of searches. *Cycle* refers to the number of iterations of the algorithm. By setting different *SN*, *Limit* and *Cycle* parameters, run the OEABC 30 times, record and analyze the fitness of the generated path.

#### 7.2.1. The Analysis of SN

**Figure 5:**
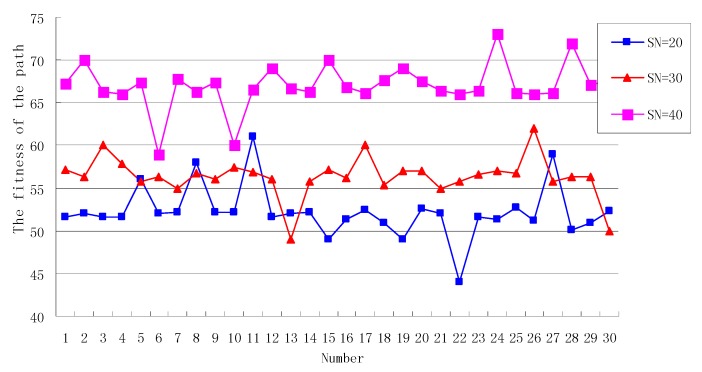
The fitness of the path when the *SN* is different.

Set *Limit* = 30, *Cycle* = 50. When the *SN* is 20, 30, 40, respectively, the fitness of the path generated by the OEABC is shown in Figure 5. As can be seen from Figure 5, When *SN* = 20, in the 30 paths generated by the algorithm, a total of four paths have a fitness change greater than 10% of the average fitness, compared with the generated paths, the ratio of paths greater than 10% of the average fitness is 13%. This is because the initial paths generated by OEABC are stochastic, and when the number of iteration *Cycle* is greater than the limit, the new path generated by the scout bee is also stochastic.

When *SN* = 30, there are three points with an amplitude of change greater than 10% of the average fitness, and compared with the generated paths, only 6% of the paths are more than 10% of the average fitness, and the variation amplitude of the generated solution tends to converge with the increase of *SN*.

When *SN* = 40, there is only one point with a variation amplitude greater than 10% of the average fitness, and compared with the generated paths, only 3% of the paths are more than 10% of the average fitness, the fluctuation range of the fitness curve becomes smaller and tends to be stable.

Therefore, the bigger the *SN* is, the higher the fitness of the path generated by the algorithm, the closer we are to the optimal solution. At the same time, it can also be seen that the path ratio of the fitness change amplitude greater than 10% of the average fitness decreases from 13% to 3%. Therefore, with the increase of *SN*, the variation amplitude of the generated solution tends to converge, and the stability of the algorithm is gradually improved. This is because *SN* becomes larger, which means that the larger the solution space is, the higher the probability of obtaining the optimal solution is. However, the increase of *SN* will lead to the synchronous increase of the overhead of the algorithm, which cannot increase the *SN* indefinitely. It is necessary to select the *SN* which is suitable for the scale of the problem.

#### 7.2.2. The Analysis of Limit

**Figure 6:**
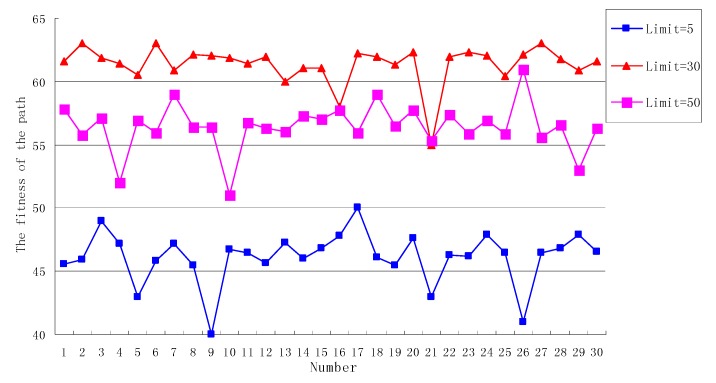
The fitness of the path when the *Limit* is different.

Set *SN* = 30, *Cycle* = 50. When the *Limit* is 5, 30, 50, respectively, the fitness of the path generated by the OEABC is shown in Figure 6.

As can be seen from Figure 6, when *Limit* = 5, the overall level of the generated solution is low, and the average fitness of the path is only 46. This is because the *Limit* value is small, meaning that it will be possible to release the scout bee multiple times to generate random paths. Randomness enhancement of the algorithm generation solution and the probability of finding the optimal solution decreases obviously because the experience obtained before cannot be fully utilized to search for a new solution.

When *Limit* = 30, although the *Limit* is not the largest, the overall level of the fitness curve is the highest, and because of the reasonable value of the *Limit*, the algorithm does not fall into a local optimum, nor is it completely dependent on stochastic generation, giving full play to the advantages of both local and global optimization of the ABC algorithm.

When *Limit* = 50, the *Limit* becomes larger, but the fitness of the generation path decreases. When the value of *Limit* is larger, the generated path can easily to fall into a local optimum, and one cannot achieve global optimization, which leads to a low level of fitness of the path, so a bigger *Limit* is not the best one, and there is a suitable *Limit* size to get a better path fitness. The size of the *Limit* needs to be proportional to the *SN*, the *Cycle* and other parameters of OEABC to avoid both complete stochasticity and local optimization.

#### 7.2.3. The Analysis of Cycle

**Figure 7:**
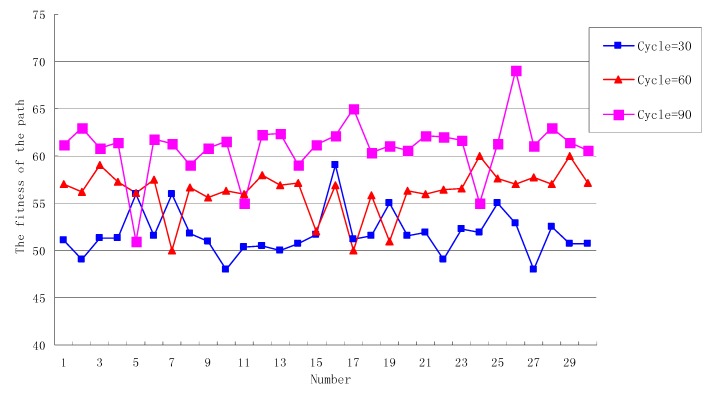
The fitness of the path when the number of *Cycle* is different.

Set *SN* = 30, *Limit* = 30, when the *Cycle* number is 30, 60, 90, respectively, the fitness of the path generated by the OEABC is shown in Figure 7.

As can be seen from Figure 7, When *Cycle* = 30, in the 30 paths generated by the algorithm, the change amplitude of fitness is greater than 10% of the average (that is, the fitness is more than 57 or less than 47), which is only the 16th path with a value of 60, merely 3% of the paths are more than 10% of the average fitness. The reason is that *Cycle* is too small and there are not enough iterations of the solution, so it is difficult to generate the path with higher fitness.

When *Cycle* = 60, compared with the previous round, the average fitness of the current round increased by nearly 10% from 52 to 58, because the *Cycle* value is large enough to enable the solution to be fully iterated.

When *Cycle* = 90, compared with *Cycle* = 60, the average fitness rose from 58 to 61, an increase of about 5%. Although the further increase of the *Cycle* enables the solution to be further optimized and the best solution 70 appears, the improvement decreases gradually. This is because the initial solution of the ABC algorithm and the solution generated by the scout bee are both stochastic, if the random solution is not good, it is difficult to generate the solution with high fitness by simply increasing the number of iterations.

Therefore, the bigger the *Cycle* is, the higher the fitness of the algorithm generating path is. It can also be seen that when the *Cycle* rises from 30 to 60, the average fitness increase is significantly greater than when the *Cycle* rises from 60 to 90. That is to say, when the *Cycle* reaches a certain value, the effect of improving path fitness will be worse and worse simply by adding to the *Cycle* number. The *Cycle* number not only maintains a proportional relationship with the *SN*, *Limit* and other parameters, but also considers the computational ability of nodes to ensure that the solutilon can be quickly iterated.

In summary, through the analysis of the *SN*, *Limit* and *Cycle* parameters, it can be seen that the fitness of the path generated by OEABC with different parameters changes greatly, which has a great impact on the performance of the OEABC. The *SN* represents the size of the bee colony, and a larger colony will be able to cover a wider range of solution space, effectively improving the probability of finding an optimal solution. Through the roulette mechanism, OEABC implements positive feedback on the path of higher fitness, by conducting the *Cycle* number of rounds of search in the neighborhood, iterate to find the path with higher fitness, but the problem is that it is easy to fall into a local optimum. The scout bee mechanism is used to solve this problem, and when a path cannot be optimized after the *Limit* iterations, it will be discarded and a random path will be generated, ensuring an opportunity to continue searching for the optimal solution globally. In the specific application scenario, selecting parameters not only considers the performance of the OEABC algorithm itself, but also should take into account the size of the body area network, the computational ability of nodes and specific requirements that need to be addressed.

### 7.3. Comparative Analysis

In order to further verify the performance of the algorithm, OEABC, the genetic algorithm [37] and ant colony [38] algorithms are used to generate the optimal energy consumption path in the above network environment. Two experiments are designed to compare and analyze the three heuristic algorithms. In Experiment 1, we compared the percentage for the appearance of optimal solution, while in Experiment 2 is the convergence rate of the algorithm.

**Experiment** **1.** *The population size and the number of iterations of the three algorithms are the same, 20 and 100, respectively, other parameter settings also choose the most commonly used settings, details as follows: OEABC: The Limit parameter is 30. Genetic Algorithm: crossover probability Pc is 0.8, mutation probability Pm is 0.2. Ant Colony Algorithm: the proving factor ALPHA is 1.0, the expected factor BETA is 2.0, the pheromone volatility coefficient ROU is 0.5.*

We run the above three algorithms 50, 100 and 150 times, respectively, then calculate the percentage for the appearance of the optimal solution. The calculation method is the number of optimal solutions divided by the total number of runs, and the results are shown in Table 7.

**Figure 8:**
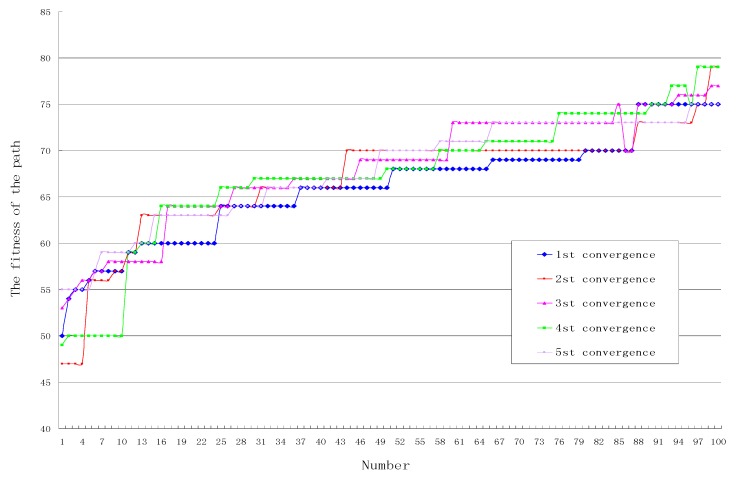
The convergence curve of the algorithm.

**Experiment** **2.** *In order to verify the convergence speed of the algorithm, in the above network environment, set the parameters SN = 30, Limit = 20, Cycle = 50, by recording the fitness of five paths generated by OEABC that form a convergence curve. The algorithm runs five times, and the corresponding convergence curve shown in Figure 8.*

The convergence rate *Cv* is calculated as:

(12) $$Cv=\frac{f(r_{opt})-f(r_{init})}{Cycle}$$

where *r_init_* is the initial path, *r_opt_* is the optimal energy consumption path.

As can be seen from Figure 8, after 50 iterations, the 1st convergence is from 50 to 75, *Cv* is 0.5; the 2th from 47 converge to 79, *Cv* is 0.64; the 3th from 53 converge to 77, *Cv* is 0.48; the 4th from 49 converge to 79, *Cv* is 0.6; the 5th from 55 converges to 75, *Cv* is 0.4, so the arithmetic average of 50 times *Cv* is 0.52. 

Comparing the convergence rates of the above three algorithms in the case of population size 20, 30, 40, the convergence rate *Cv* is calculated using Equation (12). The number of iterations are 50, the other parameters are the same as in Experiment 1. The experimental results are shown in Table 8.

Through the comparison results, we can see the OEABC has an obvious advantage in solving the problem of optimal energy consumption path of WBANs, whether the percentage for the appearance of optimal solution or the convergence rate of the algorithm are considered.

## 8. Conclusions

By considering the factors that affect the energy consumption of the WBANs, the definition of the optimal energy consumption path is given. Combined with the idea of ABC, using the method of exchanging path SA, the OEABC algorithm is designed and implemented, and the ABC is successfully applied to solve the problem of discrete solution space. The experimental results show that the OEABC can effectively solve the NP problem of the optimal energy consumption path in WBANs. Compared with the ant colony algorithm and genetic algorithm, it is further shown that the OEABC has a good performance in obtaining the optimal path of energy consumption and convergence rate.

## Figures and Tables

**Table 1:** Comparative Analysis of Different Routing Mechanisms.

Routing Protocol	Routing Mechanism	Advantage	Disadvantage	Energy Consumption
Star topology	Single Hop	Low delay	High energy consumption	High
AnyBody [17]	Cluster-Based Multi Hop	Reliable, low load	More complex	Medium
HIT [18,19]	Cluster-Based Multi Hop	Reliable, low load	More complex	Medium
WASP [20]	Cross-Layer Multi Hop	Reliable, low load	Large network delay	Medium
CICADA [21,22]	Cross-Layer Multi Hop	Reliable, low load	Large network delay	Medium
[23,24,25]	Collaborative	Low packet loss rate and low network delay	Unable to adapt to frequent network topology changes	Low

**Table 2:** Simulation Parameter Settings.

Parameter	Value
Simulation area:	100 m × 100 m
Energy model:	Generic radio energy model
Attenuation model:	Two ray
Signal transmission range:	10 m
Signal interference range:	20 m
Packet size:	512 Byte
Output queue type:	First-In First-Out (FIFO)
Channel capacity:	1 Mbit/s
Cache capacity:	50 packets

**Table 3:** The stage of initialization with five paths.

No.	Path	*η*	*C*	*E*	Fitness
1	*v*_0_, *v*_6_, *v*_19_, *v*_35_, *v*_53_, *v*_67_, *v*_88_, *v*_100_, *v*_112_, *v*_133_, *v*_140_, *v*_150_	0.57	0.63	26	46.97
2	*v*_0_, *v*_5_, *v*_28_, *v*_37_, *v*_60_, *v*_75_, *v*_84_, *v*_100_, *v*_118_, *v*_132_, *v*_138_, *v*_150_	0.49	0.56	29	45.26
3	*v*_0_, *v*_13_, *v*_28_, *v*_35_, *v*_49_, *v*_71_, *v*_81_, *v*_97_, *v*_107_, *v*_123_, *v*_136_, *v*_150_	0.67	0.48	31	57.43
4	*v*_0_, *v*_10_, *v*_23_, *v*_43_, *v*_48_, *v*_74_, *v*_85_, *v*_93_, *v*_114_, *v*_134_, *v*_139_, *v*_150_	0.68	0.45	22	58.42
5	*v*_0_, *v*_7_, *v*_27_, *v*_41_, *v*_56_, *v*_73_, *v*_90_, *v*_93_, *v*_109_, *v*_131_, *v*_149_, *v*_150_	0.49	0.44	19	49.13

**Table 4:** The stage of worker bees.

No.	Path	*η*	*C*	*E*	Fitness
1	*v*_0_, *v*_6_, *v*_19_, *v*_35_, *v*_53_, *v*_67_, *v*_88_, *v*_100_, *v*_112_, *v*_133_, *v*_140_, *v*_150_	0.63	0.61	23	50.19
2	*v*_0_, *v*_5_, *v*_28_, *v*_37_, *v*_60_, *v*_75_, *v*_84_, *v*_100_, *v*_118_, *v*_132_, *v*_138_, *v*_150_	0.49	0.56	29	45.26
3	*v*_0_, *v*_13_, *v*_28_, *v*_35_, *v*_49_, *v*_71_, *v*_81_, *v*_97_, *v*_107_, *v*_123_, *v*_136_, *v*_150_	0.67	0.48	31	57.43
4	*v*_0_, *v*_10_, *v*_23_, *v*_43_, *v*_48_, *v*_74_, *v*_85_, *v*_93_, *v*_114_, *v*_134_, *v*_139_, *v*_150_	0.69	0.43	20	59.76
5	*v*_0_, *v*_7_, *v*_27_, *v*_41_, *v*_56_, *v*_73_, *v*_90_, *v*_93_, *v*_109_, *v*_131_, *v*_149_, *v*_150_	0.49	0.44	19	49.13

**Table 5:** The stage of onlookers.

No.	Path	*η*	*C*	*E*	Fitness
1	*v*_0_, *v*_6_, *v*_19_, *v*_35_, *v*_53_, *v*_67_, *v*_88_, *v*_100_, *v*_112_, *v*_133_, *v*_140_, *v*_150_	0.63	0.61	23	50.19
2	*v*_0_, *v*_5_, *v*_28_, *v*_37_, *v*_60_, *v*_75_, *v*_84_, *v*_100_, *v*_118_, *v*_132_, *v*_138_, *v*_150_	0.51	0.56	29	46.26
3	*v*_0_, *v*_13_, *v*_28_, *v*_35_, *v*_49_, *v*_71_, *v*_81_, *v*_97_, *v*_107_, *v*_123_, *v*_136_, *v*_150_	0.66	0.45	23	57.52
4	*v*_0_, *v*_10_, *v*_23_, *v*_43_, *v*_48_, *v*_74_, *v*_85_, *v*_93_, *v*_114_, *v*_134_, *v*_139_, *v*_150_	0.69	0.43	20	59.76
5	*v*_0_, *v*_7_, *v*_27_, *v*_41_, *v*_56_, *v*_73_, *v*_90_, *v*_93_, *v*_109_, *v*_131_, *v*_149_, *v*_150_	0.49	0.44	19	49.13

**Table 6:** Results after 50 iterations.

No.	Path	*η*	*C*	*E*	Fitness
1	*v*_0_, *v*_6_, *v*_19_, *v*_35_, *v*_53_, *v*_67_, *v*_88_, *v*_100_, *v*_112_, *v*_133_, *v*_140_, *v*_150_	0.69	0.51	21	56.21
2	*v*_0_, *v*_5_, *v*_28_, *v*_37_, *v*_60_, *v*_75_, *v*_84_, *v*_100_, *v*_118_, *v*_132_, *v*_138_, *v*_150_	0.51	0.42	27	52.01
3	*v*_0_, *v*_13_, *v*_28_, *v*_35_, *v*_49_, *v*_71_, *v*_81_, *v*_97_, *v*_107_, *v*_123_, *v*_136_, *v*_150_	0.75	0.31	17	71.46
4	*v*_0_, *v*_10_, *v*_23_, *v*_43_, *v*_48_, *v*_74_, *v*_85_, *v*_93_, *v*_114_, *v*_134_, *v*_139_, *v*_150_	0.73	0.33	18	68.60
5	*v*_0_, *v*_7_, *v*_27_, *v*_41_, *v*_56_, *v*_73_, *v*_90_, *v*_93_, *v*_109_, *v*_131_, *v*_149_, *v*_150_	0.54	0.39	17	54.34

**Table 7:** The percentage for the appearance of optimal solution.

Run Times	OEABC	Genetic Algorithm	Ant Colony Algorithm
50	16.00%	14.00%	18.00%
100	15.00%	16.00%	11.00%
150	16.67%	13.33%	17.33%
Average	15.89%	14.44%	15.44%

**Table 8:** The convergence rate.

Population Size	OEABC	Genetic Algorithm	Ant Colony Algorithm
20	0.81	0.76	0.83
30	0.89	0.92	0.89
40	1.17	1.08	1.01
Average	0.96	0.92	0.91
